# Bibliometric analysis of regional Latin America's scientific output in Public Health through SCImago Journal & Country Rank

**DOI:** 10.1186/1471-2458-14-632

**Published:** 2014-06-21

**Authors:** Grisel Zacca-González, Zaida Chinchilla-Rodríguez, Benjamín Vargas-Quesada, Félix de Moya-Anegón

**Affiliations:** 1 Department of Teaching and Research, National Medical Sciences Information Centre-Infomed, 27 entre M y N, CP: 1400, Plaza, Havana, Cuba; 2 CSIC, Institute of Public Goods and Policies, Albasanz 26-28, 28037 Madrid, Spain; 3 Department of Information and Communication, University of Granada, Campus de Cartuja s/n, 18071 Granada, Spain; 4 SCImago Reseach Group, Madrid, Spain

## Abstract

**Background:**

In the greater framework of the essential functions of Public Health, our focus is on a systematic, objective, external evaluation of Latin American scientific output, to compare its publications in the area of Public Health with those of other major geographic zones. We aim to describe the regional distribution of output in Public Health, and the level of visibility and specialization, for Latin America; it can then be characterized and compared in the international context.

**Methods:**

The primary source of information was the Scopus database, using the category “Public Health, Environmental and Occupational Health”, in the period 1996–2011. Data were obtained through the portal of SCImago Journal and Country Rank. Using a set of qualitative (citation-based), quantitative (document recount) and collaborative (authors from more than one country) indicators, we derived complementary data. The methodology serves as an analytical tool for researchers and scientific policy-makers.

**Results:**

The contribution of Latin America to the arsenal of world science lies more or less midway on the international scale in terms of its output and visibility. Revealed as its greatest strengths are the high level of specialization in Public Health and the sustained growth of output. The main limitations identified were a relative decrease in collaboration and low visibility.

**Conclusions:**

Collaboration is a key factor behind the development of scientific activity in Latin America. Although this finding can be useful for formulating research policy in Latin American countries, it also underlines the need for further research into patterns of scientific communication in this region, to arrive at more specific recommendations.

## Background

In the past few decades there has been a strong accent on health research, accompanied by a noteworthy increase in funding worldwide, while demand for research into Public Health grows. There is also a heightened awareness of the political, economic, environmental and social factors that are considered determinant for a state of good health [1].

In 1999, the *Pan American Health Organization/World Health Organization* (PAHO/WHO), together with the *Centro Latinoamericano de Investigación para Sistemas de Salud* (CLAISS) and the US Center for Disease Control (CDC), launched the initiative “La Salud Pública en las Américas” in order to establish bases for a focus on fortifying Public Health in the region. This initiative aimed to arrive at a general consensus as to the concept of Public Health and its essential functions, to elaborate a method for measuring the execution of these functions and offer support for self-appraisal of the state of health in each country [2]. The essential functions were defined as the set of governmental measures needed to reach the goal of public health, and to improve, promote, protect and recover the health of the population by means of collective measures. Within these, function number 10 refers to key research for the development and application of innovative solutions in Public Health. The indicators evaluated within function 10 are: the development of research plans in Public Health; the development of the institutional research capacity; and counseling and technical support for investigating subnational levels of Public Health. In 2001 and 2002, 41 countries and territories in North, South and Central America applied the tool for evaluation of the essential functions at a national level. Some of the results at the regional level [2,3] make manifest two fundamental aspects which are, in part, the motivation behind this study.

The first of these is that function 10 presents a low level of implementation (0.35 out of 1, with a median of 0.42). The main weaknesses are the lack of national plans for research into Public Health; the lack of periodical evaluation in fulfilling the program; the inexistence of mechanisms to ensure correspondence between research and national priorities; and finally, the absence of formal and transparent mechanisms for the assignment of resources for research in many countries. The strength common to many countries is that there are tools and experts to encourage research efforts and there is sufficient health authority to develop these and utilize the results. However, it was found that just 32% of countries divulge the results of such research.

In this context, an external, systematic and objective evaluation of the scientific output of the region as a macro aggregate and its comparison worldwide could prove to be a basic analytical instrument for establishing points of reference and facilitating decision-making in the formulation of research policies related with health, the creation of useful links between research and national programs, the allotment of resources and the possibility of generating a plan for action to improve Public Health research. The information obtained about the state of the generation and transfer of knowledge in the area of Public Health would be fundamental for governments and the scientific community, and especially for decision- and policy-makers.

Given these needs and aims, the goal of the present study is to provide an objective tool regarding the research capacity of Latin America, measured and comparatively assessed in the light of scientific publications worldwide. To this end, and as part of a doctoral thesis, a series of scientometric studies were carried out to combine the results of research with the socioeconomic indicators of health at different levels of aggregation centered on Latin America and the countries that comprise it. This first contribution looks at the regional level, to contextualize the results of Latin America with the rest of the geographic regions.

More specifically, the objectives are:

• To describe the regional distribution of scientific production in Public Health, its specialization and visibility.

• To characterize Latin American scientific output in the realm of Public Health, and its position in the international context in order to establish points of reference that will serve to identify strengths and weaknesses of particular areas, in order to better orient efforts towards developing and consolidating research.

## Methods

The source of data used was the international, multidisciplinary database Scopus, through the free-access portal SCImago Country & Journal Rank (SJR) [4]. This has a better geographic and thematic coverage than other sources, meaning a better representation of science worldwide [5]. To obtain the data on worldwide population, we used the portal of the World Bank [6].

The thematic distribution analyzed corresponded to the category *Public Health, Environmental and Occupational Health* in the area Medicine, for the period 1996–2011. In the geographic distribution we worked at the macro level with the world aggregate, together with ten regional aggregates (Latin America, North America, Western Europe, Eastern Europe, Northern Africa, Central Africa, Southern Africa, Middle East, Asia, and the Pacific).

The indicators used were:

$$\mathrm{TSI}=\frac{\mathrm{Ndoc}\;\mathrm{Public}\;\mathrm{Health}\;\left(\mathrm{country}\right)\;÷\;\mathrm{Ndoc}\;\left(\mathrm{country}\right)\;}{\mathrm{Ndoc}\;\mathrm{Public}\;\mathrm{Health}\;\left(\mathrm{world}\right)\;÷\;\mathrm{Ndoc}\;\left(\mathrm{world}\right)\;}$$

• Total number of documents published between 1996 and 2011 (Ndoc)

• Number of citable documents: articles, reviews and conference proceedings (Ndocc)

• Rate of growth calculated by the difference (%) between the number of works in relation with the previous period (RG)

• Number of documents per one million inhabitants

• Relative Specialization Index (RSI) or Relative Activity Index: this measure indicates whether a country has a relative higher or lower share in world publication in Public Health than its overall share in world total publication. It is calculate based on the thematic Specialization index (TSI). This indicator is closely related to the so called Activity Index (AI) long used in bibliometrics, which, in turn, is a version of the economists’ Comparative Advantage Index [7,8].

$\mathrm{RSI}=\frac{\mathrm{TSI}-1}{\mathrm{TSI}+1}$; RSI can take values in the range -1 and 1, 0 correspond to the world average, RSI < 0 indicates a lower that average, RSI > 0 a higher than the average activity.

• Percentage of international collaboration, assessed by the number of works involving authors from more than one country.

• Number of times cited in any type of document (Cites)

• Number of self-citations by authors of the same region (Autocit)

• Average number of citations per document (Cpd)

• Percentage of documents cited (% Ndoc-cit)

• H-index, considering H as the number of documents of a region obtaining at least H citations.

Finally, we analyzed the correlation coefficient of the Spearman range applied to the indicators by countries.

## Results

### Volume and evolution of the regional scientific output in Public Health

**Figure 1:**
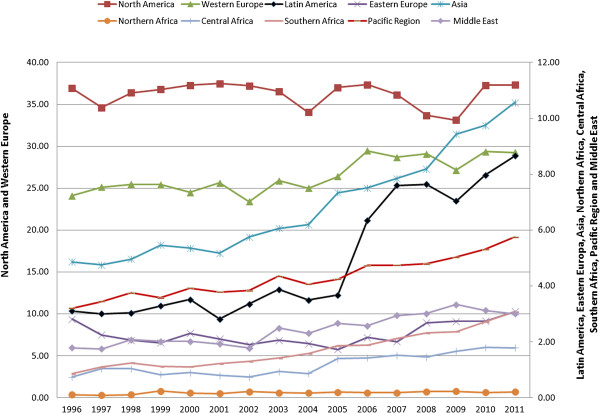
**Regional breakdown of scientific output in Public Health.** Scopus 1996–2011. Left (Table) shows percentage of contribution to world output, rate of growth, and number of documents in Public Health per one million inhabitants. Right (chart) shows annual evolution of percentage of documents in Public Health by regions with respect to total worldwide. Source: SCImago Journal & Country Rank, elaborated by the authors based on Scopus data.

Taking as reference the world domain, the results show that 27.23% of scientific output in the Scopus database pertains to the area of Medicine, with a total of 7,015,153 documents. Of these, 313,543 come under the category of Public Health, thus representing 1.22% of world output and 4.47% of publications in Medicine. Both Public Health and Medicine maintain a trend of linear growth over a similar period of time, slightly less than the aggregate of world science. According to the SJR, in the category of Public Health there were 13,234 documents published in the year 1996, and 29,924 in 2011, which is a growth rate of over 126% --approximately 55 percentage points more than the rate of growth of Medicine itself (70.9%).The regional distribution of these results is not homogeneous (Figure 1). A look at the relative contribution of each region to worldwide output in Public Health shows that North America and Western Europe produce more than 60% of the world aggregate, followed by Asia and Latin America. The African regions have the most limited participation in the world aggregate, altogether not reaching 4%.

**Figure 2:**
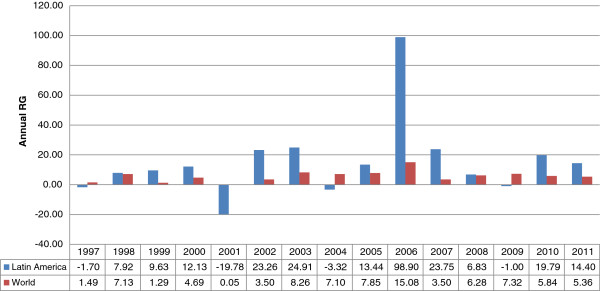
**Evolution of the annual rate of growth in Latin America and the world.** Source: SCImago Journal & Country Rank, based on Scopus data.

All the regions underwent an increase in scientific production, though growth was greater in Southern Africa and Latin America than in Central Africa and Asia. North America showed the smallest rate of growth, while having the greatest number of documents: though the gross number of publications rises, their relative weight on the worldwide scale is reduced. The contribution of the other regions increases between 2001 and 2005, except for Eastern Europe, where a relative increase is seen instead for the period 2006 to 2011.Figure 1 displays the percentage-wise evolution of the amount of output by regions with respect to worldwide production. Roughly half of all the documents came out in the past five years. Most aggregates show an irregular evolution; for instance, North America is seen to rise and fall in output. Latin America and the Pacific region had similar volumes up to 2005, but in 2006, Latin American scientific production surpassed that of the Pacific by 368 documents, and in 2011 the difference amounted to 869 publications. In the period as a whole, world output grew 126% (Figure 2), while that of Latin America climbed 530%, with slight drops in the years 1997, 2001, 2004 and 2009 offset by a sharp increase between 2005 and 2007.All the regions exhibited an increase in the indicator “number of documents per million inhabitants” in the period of study. The greatest value was found in the Pacific in the year 2010, followed by North America and Western Europe. At the beginning of the period, Latin American publication did not reach 1 document per one million inhabitants, whereas in 2010 it attained 3.85. This considerable increase moves Latin America from sixth position to fourth in the ranking by this indicator. Asia, taking fourth place in terms of scientific output, goes from last place to ninth in documents per one million inhabitants. Eastern Europe, Middle East and all three African regions are very low in this indicator (Figure 1).

### Scientific output and patterns of specialization in Public Health, by country

In the previous section, the research activity has been analyzed from the point of view of the volume of production, both in absolute and percentage terms. But to determine the thematic profiles that do not necessarily have to match the results obtained, domestic production relativized with respect to international.

We use a relative indicator, the Relative Specialisation/Activity Index that indicates whether a country has a relatively higher or lower share in world publications in Public Health than its overall share in world total publications. For each country, the graphic presents four lines related to the years 2003, 2007 and 2011 that show the evolution of indicator, and the fourth line is the referent and represents the world. In this manner, is easy to know if the indicator is above or below the world average and therefore if the country is more or less specialized in Public Health. In this scenario, a comparison of the world output in science in the framework of thematic specialization highlights the relative achievements of regions and countries with regard to international output.

**Figure 3:**
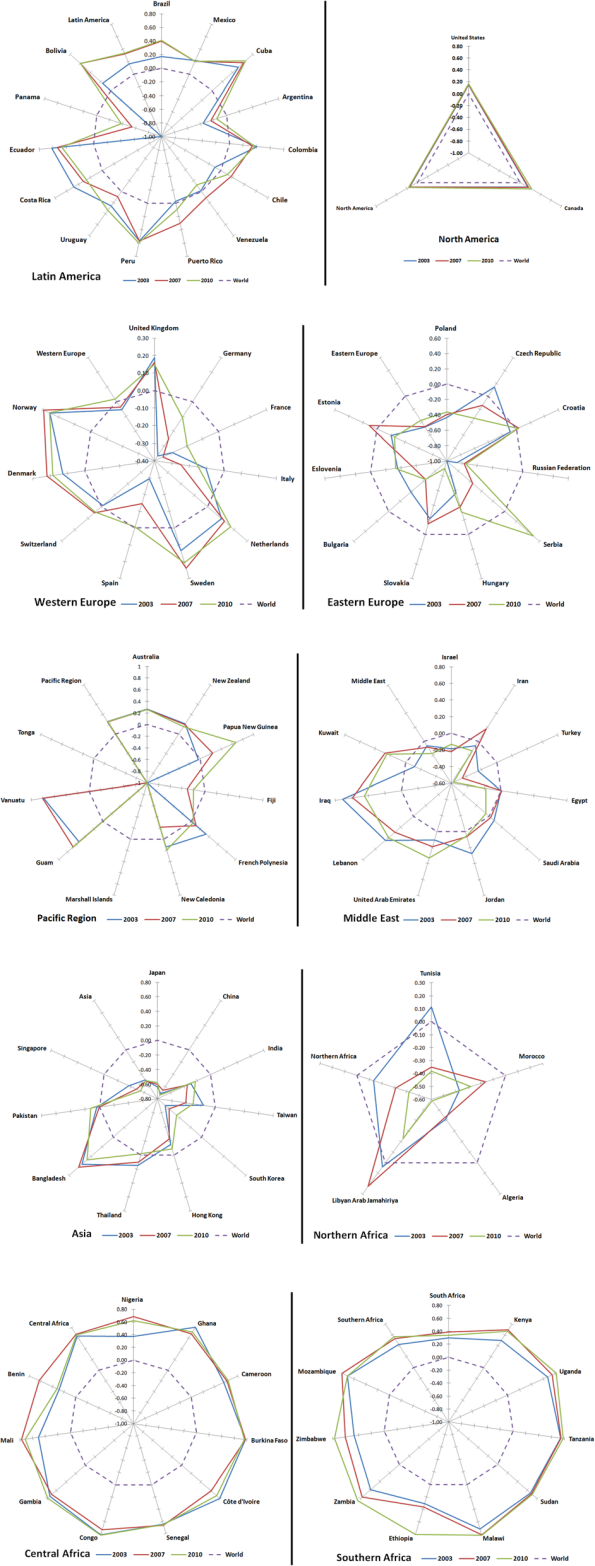
**Relative specialization index by region.** Scopus 2003, 2007 and 2010. Source: SCImago Journal & Country Rank, with Scopus data, elaborated by the authors.

Table 1 displays the five countries with greatest scientific production in each region, their position in the world ranking of 213 countries with at least one document published in the category of Public Health, and the place occupied by each aggregate in the ranking of Relative Specialization Index (RSI) for the period of study (Figure 3).

**Table 1:** Countries with the higher output in Public Health in each region

**Country**	**% Ndoc**	**Ranking Ndoc**	**Ranking RSI**	**Country**	**% Ndoc**	**Ranking Ndoc**	**Ranking RSI**
**Latin America** (42 countries)				**Northern Africa** (4 countries)			
Brazil	3.17	6	98	Tunisia	0.08	70	176
Mexico	0.82	20	130	Marocco	0.07	80	170
Cuba	0.31	35	70	Argelia	0.02	110	201
Colombia	0.26	38	84	Libyan Arab Jamahiriya	0.02	119	105
Argentina	0.26	40	178	**Central Africa** (23 countries)			
**North America** (2 countries)				Nigeria	0.60	25	54
USA	32.19	1	135	Ghana	0.13	56	34
Canada	4.77	3	133	Camaroon	0.10	60	61
**Western Europe** (25 countries)				Burkina Faso	0.10	63	13
United Kingdom	8.99	2	134	Senegal	0.08	73	50
Germany	3.84	5	177	**South Africa** (26 countries)			
France	2.68	7	184	South Africa (26 countries)	0.78	21	104
Italy	2.55	8	169	Kenya	0.25	41	53
Netherlands	2.32	9	142	Uganda	0.22	45	10
**Eastern Europe** (23 countries)				Tanzania	0.20	49	25
Poland	0.55	27	197	Ethopia	0.12	57	51
Czech Republic	0.45	29	165	**Pacific Region** (17 countries)			
Croatia	0.26	39	137	Australia	3.89	4	112
Serbia	0.25	42	83	New Zealand	0.64	23	129
Russian Federation	0.22	44	211	Papua New Guinea	0.02	117	88
**Asia** (30 countries)				Fiji	0.01	137	103
Japan	1.55	12	203	French Polinesya	0.01	152	107
China	1.55	13	208	**Middle East** (16 countries)			
India	1.40	15	188	Iran	0.62	24	154
Taiwan	0.73	22	191	Israel	0.57	26	171
South Korea	0.5	28	202	Turkey	0.40	30	199
				Egyt	0.28	36	159
				Saudi Arabia	0.20	50	145

Source: SCImago Journal & Country Rank, with Scopus data, elaborated by the author.

Within Latin America, Brazil heads the ranking of countries, with a 3.17% contribution to output overall, and it is sixth worldwide. It is followed by Mexico, in position 20, and after a jump in the number of documents, Cuba, Colombia and Argentina appear in positions 35, 38 and 40, respectively. The greatest strengths measured in thematic specialization are shown by Cuba, Colombia and Brazil, among the countries with the most scientific output, and Peru, Ecuador and Bolivia with the least. The region showed greater specialization in Public Health than the world figure of reference, particularly between 2007 and 2010. The weakest countries were Argentina and Panama.

In North America, the US and Canada occupy positions 1 and 3 in the ranking by volume of output in Public Health. The United States contributes with over 30% of total documents. In thematic specialization the US and Canada take positions 135 and 133, respectively, despite having a RSI above the world average, as does the regional aggregate.

Western Europe has a privileged position owing to the United Kingdom, Germany, France, Italy and the Netherlands, respectively in positions 2, 5, 7, 8 and 9 worldwide. The other countries of Western Europe are well situated. As a region, it surpassed the world average in the year 2010 alone. The strongest RSIs are seen in the UK and Netherlands.

Eastern Europe has countries positioned from 27 to 44 in the ranking by number of documents. Both regionally and on the national level, they are below the world average in thematic specialization except for the Czech Republic in 2003, Estonia in 2007 and Serbia in 2010.

In Asia, Japan has the most output, followed by China and India. In terms of thematic specialization, the Asian countries occupy the final positions in the ranking, and the regional aggregate is below the world average. The countries best situated in Public Health output are Bangladesh, Thailand and Pakistan.

Northern Africa’s four counties are ranked between 70 and 119. The region is well below the world average in RSI. The best situated African countries are Nigeria and South Africa. Both the regions and the component countries are above the world reference in terms of thematic specialization. Indeed, the African countries occupy the top positions in the RSI ranking, and most are above the world reference, yet they have very low levels of scientific output. Central Africa showed a balanced yield in output and specialization.

In the Pacific region, Australia and New Zealand have the greatest volume of documents, and occupy places 4 and 23 of the world ranking, respectively. The region overall is above the world average in specialization.

The five countries of the Middle East with most scientific output can be found in positions 24 to 50. The regional aggregate is consistently below the world average in thematic specialization, throughout the period of study.

### International collaboration in Public Health research/output

**Figure 4:**
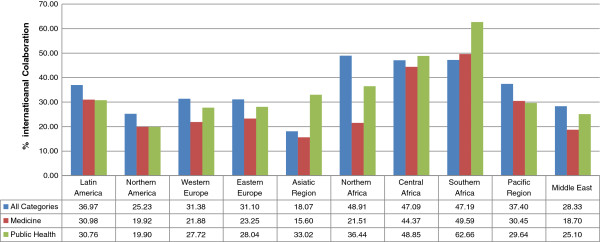
**International collaboration in Public Health, Medicine and all the categories by region.** Source: SCImago Journal & Country Rank, with Scopus data, elaborated by the authors.

The pattern of communication involving Public Health by regions is complemented with the analysis of collaboration, with reference to international collaborative efforts both in all scientific fields, on the one hand, and in Medicine and Public Health, on the other (Figure 4). During the period analyzed, the African regions presented the greatest values for international collaboration (nearly 50%), whereas Asia, followed by North America and the Middle East, have the lower percentages of internationalization in all fields.

Comparison of these global percentages with those of area and thematic category show the European regions and North America to have the highest rates of internationalization in all fields. On the other hand, collaborative research in Public Health is seen to be greater than in Medicine as a whole in Africa, Europe, Middle East and Asia. It is notable that in Asia there are big differences in international collaboration among countries in the field of Public Health. These differences are a lot higher than those found both in Medicine and in the rest of the scientific fields.

In Latin America, the figures are fairly homogeneous. Over 30% of Latin American documents in Medicine and Public Health involved international participation, a rate reflecting that of overall collaboration. In 1996, 35.8% of output was collaborative, dropping to just 25.4% in 2011; in contrast, collaboration rose in all other regions.

### Visibility of Latin American scientific output in Public Health

**Figure 5:**
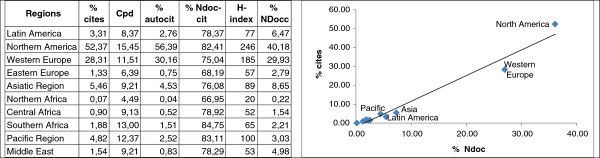
**Citation indicators by region.** Left. Percentage of citations worldwide, citations by document, percentage of documents cited, self-cited and citable in Public Health, by regions. Right. Correlation between percentage of documents and percentage of citations, with respect to world total, by region. Source: SCImago Journal & Country Rank, with Scopus data, elaborated by the authors.

As a rule of thumb, there is a high correlation between output and citation, confirmed with our results. Figure 5 displays the percentage of scientific output in the x axis, and the percentage of citation in axis y; a high correlation of R^2^ = 0.97 was obtained. That is, the greater the volume of output, the greater the likelihood of being cited. Yet we should stress that the citable production in this study included articles, reviews and conference acts. In Africa and the Pacific, there is a noteworthy gap between the figures for total output and cited output.Figure 5 offers a comparison of the rates of production and of citation. North America receives over 50% of all the citations in Public Health, 40% of citable output worldwide, and 36% of world output. Meanwhile, Latin America harvests only 3.3% of world citations, a lesser value in light of the citable documents (6.47%) and total documents (5.47%). Except for the Pacific, we see a trend of greater participation in Public Health than percentage of citations received. The African values are again low for both indicators. Medicine received 28.3% of the total citations worldwide, whereas Public Health took in 3.87% of those in the area of Medicine, and 1.47% of the global citation figure.A closer look at the indicator “citations per document” (Figure 5) can be useful for appraising the volumes on a macro level. The visibility of Public Health is less than that of Medicine (respective means of 9.91 and 10.65 citations per document). North America is the most visible region in Public Health, in agreement with the volume of its citable output. Latin America, along with Asia, lies well below the world average, and Northern Africa is last of all for this indicator. These results are conditioned by the percentages of cited documents, which are over 80% in the cases of North America, Southern Africa and the Pacific. Auto-citation stands as roughly one-fourth of total citation in all the aggregates. The relative peak in this indicator, detected between 2003 and 2006, appears proportional to the number of citations.Finally, the H index is a measurement of impact used by evaluation and financing agencies. It is not valid as an indicator in the study of regional aggregates, yet it serves to highlight our results regarding visibility. North America leads the ranking with a value of 246, followed by Western Europe, the Pacific, and Asia. The H index of Latin America is 77 in Public Health, in fifth place (Figure 5). In Medicine, the H-index ranges from 872 to 69. As it was the case with Public Health, North America has the higher value while North Africa has the lower value.

### Correlation between indicators of output, specialization, and citation

In order to discover common patterns in the regional output of Public Health documents, we calculated the correlation coefficients of the Spearman range in view of the positions that the countries occupied on the rankings by the different indicators (Table 2). No clear relationships emerged, however, given the macro level of aggregation. All the indicators show some degree of correlation, yet intensity varies. The association between the number of total documents and that of citable documents is weak, probably due to inherent properties of the indicator, which is no longer “complete” in the final years of the period; that is, it is expected that these documents will continue to be cited for some years beyond our study period. The RSI has a weak negative correlation with the indicators of output and citation, meaning that no association can be established between volume and specialization, or between specialization and visibility.

**Table 2:** Coefficient of Spearman range correlation among indicators

**Indicators**	**Ndoc**	**Ndocc**	**RSI**	**Cites**	**Autocit**	**Cpd**	**H index**
Ndoc	1.00	0.972*	-0.258*	0.950*	0.952*	0.420*	0.939*
Ndocc	0.972*	1.00	-0.257*	0.978*	0.980*	0.448*	0.967*
RSI	-0.258*	-0.257*	1.00	-0.210*	-0.222*	0.132	-0.193*
Cites	0.950*	0.978*	-0.210*	1.00	0.985*	0.589*	0.993*
Autocit	0.952*	0.980*	-0.222*	0.985*	1.00	0.527*	0.978*
Cpd	0.420*	0.448*	0.132	0.589*	0.527*	1.00	0.590*
H index	0.939*	0.967*	-0.193*	0.993*	0.978*	0.590*	1.00

*Indicates significant bilateral correlation at a level of 0.01.

## Discussion

### Scientific output by regions, representativity and specialization in Public Health

The regional distribution of the output in Public Health coincides with the pattern of communication in world scientific production in general [4,9,10], where North America, Western Europe and Asia contributed over 80% of output overall. A breakdown by counties shows the top producers to be the US, the UK, Canada, Australia and Germany, similar to the findings of Navarro and Martin [11], who studied only original articles according to the Science Citation Index-Expanded (SCI-EXPANDED) and the Social Science Citation Index (SSCI); although their ranking was similar, France took fifth place in their results. In Latin America, Brazil and Mexico were seen to be substantial producers in Public Health.

These studies point to a series of factors that determine the scientific dynamics of regions nowadays. For one, there is a growing volume of production in certain Asian countries, China foremost among them, due to an increase in funds for R + D by the Chinese government and the highly qualified human resources [12]. In 2006, China was the second country in number of documents according to the expanded SCI-Web version [13]. Also important is the emergence of South Korea, Brazil, India, the Middle East, Southeast Asia and Northern Africa [14]; the expansion of the European Union, and the comparatively high yet stagnant US output, perhaps having reached its peak [9,10,13]. The appearance of the Scopus database and the greater thematic, geographic and linguistic coverage may be additional factors [5,15].

In the case of Public Health, rising output is found in regions such as Africa, Latin America and Asia, where output was previously very low. Contrariwise, in North America, where production was quite high, a slightly downward trend is seen.

Western Europe in general makes an important contribution to Public Health research. Its volume is similar to that of the US, though they are dissimilar in visibility. The growth of science almost certainly has to do with the support lent by the European Commission for Research since the 1970s [16,17]. Growth is not homogeneous, however, and variations suggest different traditions as well as diverse levels of investment.

Eastern Europe, meanwhile, shows results similar to those reported for the period 1990–2009 by Karamourzov in 2012, regarding the independent scientific development of CIS countries (Commonwealth of Independent States). Despite the political transition and economic recovery of the past two decades, none of these Eastern countries has secured a leading position in the scientific arena. Problems for development may stem from the substantial structural changes of the 1990s, a decrease in the scientific population, and the publication of primary studies in national journals or in the Russian language, not included in international indexes [18].

In turn, the growing output in Africa may be traced to a strategic fight against poverty. The GNP of most African states increased between 2002 and 2008, though it is still low and hampers investment in science, technology and innovation. South Africa is the only country approaching 1% investment of GNP; it’s Gross Budget for Research and Development (GBID) in 2007 was 0.93% [19].

Normalizing the number of documents by number of inhabitants reveals the Pacific region to have intensive activity in Public Health, along with North America and Western Europe. Similar results were described by authors Rahman and Fukui, who compared the regions using numbers of biomedical publications per one million inhabitants in the period 1990–2000 [20].

The study by Falagas et al., covering three biomedical areas including Public Health, showed Latin America to be second to last in the number of documents, followed only by Africa. Standardizing this indicator for total inhabitants leads to the same results, and slightly better ones when adjusted by GNP [21]. Another study using subcategories Preventive Medicine, Occupational and Environmental Medicine; Epidemiology; and Public Health revealed that Latin America produced 1.5%, 1.7% and 1%, respectively, during the period 1995–2003, according to the Journal Citation Report (JCR) database of the ISI [22].

In recent years, Latin America has undergone considerable improvement in Public Health output. A number of factors have been influential: a greater awareness of the need to foment research, a focus on Public Health problems, and efforts to improve the quality of life of the population overall. Despite more funds for Public Health, the number of researchers is low, and many researchers go abroad in search of better opportunities for training and producing science [1,19,23,24].

Another factor is the entry of journals in the SciELO database (Scientific Electronic Library Online) in Scopus, which could have contributed to the growth of the Latin American aggregate. Since its incorporation, it was intended to enhance the visibility of Latin American scientific output [25-27]. Brazil is emerging in economic terms as well as research efforts, and its exponential growth since the 1990s has been underlined in many studies, characterized by publication largely in national journals, while having lower rates of international collaboration than the rest of the Latin American countries [28-30].

### International collaboration and visibility

At present, enhanced collaboration is desirable at all levels and in all productive sectors, including scientific output. Policy holds that collaboration favors sustainable development, which is the foundation for the socioeconomic independence of developing countries, and heightens research visibility [31].

Latin America follows the international pattern of fomenting scientific cooperation among countries, linked with greater quality and fortified scientific capacity [32]. Yet we spot an opposite, downward trend in Public Health, evidenced by countries such as Brazil and Cuba [30]. Clearly, the relations among research institutions, universities and the productive sector could be improved [23].

The African regions, on the other hand, manifest growing collaboration. This has implications for their visibility; but most likely reflects a certain dependence upon collaborating associates from countries strong in science rather than leadership per se [33]. Developing regions should actively learn and cooperate with economic power to enhance their scientific research level and change their position in information dissemination [34].

The low level of collaboration found for Asia coincides with previous studies where the region was confirmed as a nucleus of Public Health research [11]. China, the main producer, does not increase in citation at the same rate as it does in Public Health output, a trend pointed out by a previous global analysis [12]. In contrast, North America, with modest collaboration, is a world leader in terms of output and visibility in the field.

Regarding auto-citation, North America and Western Europe more clearly rely on the wealth of knowledge they themselves produce or that of nearby geographic areas. This would explain the concentration of impact in the most productive regions [35]. The high values for self-citation are logical in view of the high volume of citable output harvested in these two regions, with ample scientific communities contributing to the mainstream.

### Correlation between bibliometric indicators

The analysis of correlation between/among indicators suggests that a greater volume of scientific output is accompanied by greater visibility and lesser thematic specialization. However, this study is just an initial venture toward the domain of Public Health at the regional level. Specific characteristics or trends of each country may emerge in further studies, clarifying the associations between volume, visibility and specialization of a country.

## Conclusions

World scientific output in Public Health represents some 5% of the total production in Medicine, and is less visible. The regional distribution coincides with that of science in general: North America and Western Europe are the most productive regions as well as the most visible ones, the Pacific region is characterized by a high impact and degree of specialization, Asia has noteworthy sustained growth, Eastern Europe and the Middle East are low in the rankings for quantity and quality, and the African regions are the least productive of all; however, they have a high level of specialization and more articles in collaboration.

The Latin American contribution to the world arsenal of science can be considered scanty. The greatest strengths are its high level of specialization in Public Health and the sustained growth of its output. It has risen to fifth position in the international ranking. Together with Asia, Latin America is the region showing the fastest development in research over the past 16 years. Within Latin America, Brazil is the top producer, and Brazil, Cuba and Colombia have high levels of specialization in Public Health.

Among the weaknesses, we may mention the decreasing international collaboration despite the internationalization of science through cooperation, which is seen as a factor encouraging scientific development in Latin America. Notwithstanding, the lower volume of documents in international collaboration does not mean a decrease in the number of participating countries. Further analysis of each specific country would be necessary to determine whether this is a generalized trend, or rather a phenomenon of just some countries. Another weakness is that the increase in the number of articles is not yet reflected in the visibility in the scientific output in terms of citation, although it is reflected in the greater international presence in the first reference of the world scientific literature.

The results of this paper may be viewed as a diagnostic tool for measuring Latin America’s research capacity, and also serve to infer its potential through comparison with other world regions. Such information is necessary to monitor the essential function of research for the development and application of innovative solutions in the domain of Public Health.

Future work will lead us to a combined study of socio-economic indicators (investment and human resources) and bibliometrics of the main Latin American countries in order to enrich the analysis of results with the research efforts that each country dedicates to an area with vast social and economic repercussions: Public Health.

## Competing interests

The authors declare that they have no competing interests.

## Authors’ contributions

GZ-G gathered data from source, carried out the analysis of data and drafted the manuscript, ZC-R conceived of the study, and participated in its design and coordination and helped to draft the manuscript, BV-Q – conceived of the study, and participated in the design and performed the statistical analysis, FM-A carried out the main bibliometric indicators and the development of source of information used. All authors read and approved the final manuscript.

## Pre-publication history

The pre-publication history for this paper can be accessed here:

http://www.biomedcentral.com/1471-2458/14/632/prepub
